# Pilot study employing heart rate variability biofeedback training to decrease anxiety in patients with eating disorders

**DOI:** 10.1186/2050-2974-2-17

**Published:** 2014-06-03

**Authors:** Barbara Scolnick, David I Mostofsky, Robert J Keane

**Affiliations:** 1 Department of Psychology, Boston University, 64 Cummington Street, Boston, MA 02215, USA; 2 Clinical Operations, Walden Behavioral Care, 9 Hope Avenue, Waltham, MA 024533-2711, USA

## Abstract

Heart rate variability (HRV) biofeedback, a technique which encourages slow meditative breathing, was offered to 25 in-patients with various eating disorder diagnoses-anorexia nervosa, bulimia nervosa and binge eating disorder. We found that this modality had no serious side effects, and was subjectively useful to most participants. An enhanced ability to generate highly coherent HRV patterns in patients with recent onset anorexia nervosa was observed.

## Dear Editor

We were interested in whether heart rate variability biofeedback training, which resembles yoga and mindfulness meditation, would be helpful in decreasing anxiety for patients with eating disorders as it has been found to be useful in patients with depression, and post traumatic stress disorder [1,2]. However, due to the unique physiological changes accompanying eating disorders, we had concerns that it might precipitate syncope or paradoxically increase anxiety, so we performed a pilot study in an inpatient unit.

Heart rate variability (HRV) refers to the normal phenomena in which time between one beat of the heart and the subsequent beat is not absolutely uniform, but shows variability. A great deal of the variability is determined by respiration—the pulse speeds up with inspiration and slows down with expiration. It has been appreciated for over one hundred years that heightened variability, measured by palpating the pulse for several minutes, is a sign of physical fitness, youth, and general good health [3].

The advent of digital signal processing in the 1970s led to more sophisticated statistical descriptions of this phenomena and an explosion of research on how HRV changes in various diseases and states. In addition to simply measuring beat-to-beat intervals over time, the ensuing wave pattern could be partitioned into frequency components, in real time. Pharmacological studies, suggested that most of the higher frequency variability was due to vagal activity, while the lower frequency was due to sympathetic activation [4]. Unfortunately, while autonomic tone clearly is reflected in heart rate variability, it is only an indirect assessment.

While most disease states from diabetes, to congestive heart failure, to depression, and chronic pain are associated with decreased total HRV and specifically decreases in higher frequencies, suggesting a relative lack of vagal tone, anorexia nervosa has been the exception [5]. Although there is no consensus, most studies show that patients with anorexia nervosa have elevated HRV compared to normal controls [6]. When only adolescents with anorexia nervosa-restriction type are studied, results show consistent and more extreme elevation of HRV [7,8]. The results with bulimia are mixed, although most studies report elevated total HRV [9]. During the refeeding process, the total HRV tends to decrease, even before other parameters such as leptin levels or body mass index change significantly [10].

Heart rate variability biofeedback training (HRV BF) is a technique that essentially borrows the breathing aspects of Eastern meditation techniques, and presents the information as a personalized digital display [11]. A pulse monitor or EKG lead is placed on the subject, and the resultant pulse or EKG tracing, and inter-beat-intervals are displayed on a computer monitor. Slow breathing exaggerates HRV, and at a breathing rate of approximately 6 breaths/minute, the pattern becomes visible larger, and forms a sine wave. This has been termed “resonance” or “coherence”, and can be measured mathematically and appreciated visually [12]. The patient is encouraged to generate this pattern by slow breathing and evoking peaceful feelings.

There is agreement that the resonant pattern is mediated primarily by enhanced vagally mediated “brake and release” stimulation of the heart rate, in synch with respiration. Although there are differing opinions, some practitioners think it also reflects and enhances a state of mental calmness [13]. All agree that, at the minimum, HRV BF encourages slow breathing.

Because patients with eating disorders, especially anorexia nervosa uniquely have elevated HRV in the higher frequencies, our concern was that we might precipitate syncope or a sense of ill-ease by further encouraging vagal tone. We, thus, performed this pilot in a unit where there was 24 hour monitoring of the patients.

## Study design

This study was approved by the Boston University Institutional Review Board; we obtaining signed informed consent from adults and when the participant was younger than 18, from the adolescent and both parents. HRV biofeedback was offered to 25 patients; 1 immediately refused, (the pulse meter was irritating), and 24 agreed to participate. The demographics are summarized in Table 1. We used a commercially available product emWave-pro by Heart Math®, and a Mac Book Pro computer; sessions were usually 10 minutes, occurred daily or every other day for as long as the participant was inpatient, up to 12 sessions. The technician attached the pulse meter to the participant’s ear lobe, and reviewed the computer screen which displayed the inter-beat interval time series, and percent coherence. A bong sounded every five seconds, the pitch reflecting whether coherence was increasing or decreasing. The technician encouraged the patient to breathe at 6 breaths/minute, and try to invoke a sense of peace. Frequently the patients would chose to think about, a beloved pet, or aspects of nature, such as ocean waves, or sunsets.

## Results

There were no serious side effects. Only one patient, a 24 year old woman with anorexia nervosa-restricting type and comorbid bipolar disorder, felt faint while doing the feedback. Her vital signs were stable, but when it recurred during the next session, she was advised not to continue.

Four participants asked to leave the study early because they were feeling no benefit. Three were young teens that were ill for less than one year and insisted there was no problem with their eating, stating they were eating “healthy” and enjoyed being “fit”. The other was a 17 year old girl who also generated 100% coherence on her first attempt, completed 10 sessions and was enthusiastic about the technique, reporting it was the most helpful aspect of her hospitalization. By session 10 she had gained 10 pounds, had come to accept her eating was a problem, had more difficulty generating a coherent pattern, and felt frustrated by the technique.

The remaining 19 patients continued to take part in the study until discharge.

Seventeen patients completed 5 sessions and filled out a self-administered questionnaire. In response to the statement; “Biofeedback decreased my anxiety” 47% reported it was strongly true, 35% reported somewhat true, and 17% were neutral.

A surprising result was the differences in the ease of generating coherence, which generally correlated with youth, and how this ease did not necessarily correlate with subjective benefit. As shown in Table 1, most young adolescents were able to achieve 100% coherent pattern in the first training session. Many seemed to enjoy what one teen labeled, “my unique ability”, but this did not necessarily translate to reports of decreased anxiety. Those who were most enthusiastic about the biofeedback were patients in their 20's who uniformly considered their eating disorders unwanted illnesses and were trying to attain recovery. None of these patients generated coherent patterns immediately but eventually many “got the hang of it”, and could generate a sine wave by both slowing their breath and invoking comforting thoughts. The five patients who were older than 30 struggled to generate coherence, yet they reported the slow breathing was calming.

**Table 1:** Demographics, & percent coherence attained

**Age**	**13-15**	**16-18**			**19-25**		**26-29**		**30-55**	
**Diagnosis**	**ANR**	**ANR**	**AN BP**	**BN**	**ANR**	**BN**	**ANR**	**BN**	**ANR**	**BED**
Number of participants	4	4	1	1	5	1	2	1	4	1
Percent coherence attained-range	86-100	93-98	65	100	67-100	100	77-98	85	0-50	50
Number of participants who attained 100% coherence	3	0	0	1	1	1	0	0	0	0
Session that 100% coherence was achieved	1	--	--	7	4	4	--	--	--	--

ANR anorexia nervosa restricting type.

ANBP anorexia nervosa binge/purge type.

BN bulimia nervosa.

BED binge eating disorder.

## Implications

As far as we know, this is the first report of HRV BF in patients with eating disorders, although several studies, of nonclinical populations have demonstrated efficacy of HRV BF on lessening cravings for chocolate [14,15].

The main finding from this pilot study is that HRV BF training is safe in this population. It can be considered an adjunctive treatment akin to meditation or yoga, which is beneficial to some individuals more than others.

We were intrigued by the ease adolescents with anorexia nervosa demonstrated in generating large coherent sine waves, which is consistent with the previously reported baseline elevation in HRV in this population compared to normal controls. Since elevation of HRV is a sign of physical fitness and health, this may simply be a reflection of the exercise habits of many patients with anorexia nervosa.

One of the vexing paradoxes of anorexia nervosa is that prior to onset and diagnosis, an adolescent can appear a model citizen; intent on exercising, reasonable concerned about eating “healthy”, and we can presume displaying a “healthy” robust HRV. The mechanism that occurs transforming this state into one of self-driven starvation remains a deep mystery. If a substantial difference exists between HRV in healthy athletic adolescents, as compared to those with anorexia nervosa, it might be a biomarker for this disease.

## Competing interests

The authors declare they have no competing interests.

## Authors’ contributions

BS carried out the technical aspects of the study. DM noted the theory regarding the biofeedback. RJK monitored the clinical status of the patients. All authors read and approved the final manuscript.
